# Strong correlation of total phenotypic resistance of samples from household environments and the prevalence of class 1 integrons suggests for the use of the relative prevalence of *intI1* as a screening tool for multi-resistance

**DOI:** 10.1371/journal.pone.0218277

**Published:** 2019-06-13

**Authors:** R. Lucassen, L. Rehberg, M. Heyden, D. Bockmühl

**Affiliations:** Hochschule Rhein-Waal, Faculty of Life Sciences, Kleve, Germany; Centre for Ecology and Hydrology, UNITED KINGDOM

## Abstract

One of the biggest challenges of health care systems worldwide is the increasing number of pathogenic bacteria resistant to a growing number of antibiotics. In this respect, class 1 integrons which are part of mobile genetic elements can confer several phenotypes including resistance to a broad range of antibiotic classes, heavy metals and biocides. They are linked to common resistance genes and have penetrated pathogenic and commensal bacteria likewise. Therefore its relative prevalence can be a proxy for antimicrobial resistance and anthropogenic pollution. Household environments are areas with a high influx of bacteria from humans, animals and foods, and biocides and detergents are frequently used. In this study we aimed to investigate the relative prevalence of class 1 integrons in household environments, in relation to the number of antibiotic and benzalkonium chloride resistant phenotypes of a sample point, for the validation of the relative prevalence of class 1 integrons as a screening tool for multi-resistance. Kitchen sink and bathroom sink U-bends, dishwasher, washing machines and toothbrushes of 28 households were probed. Copies /mL of class 1 integron integrase gene and 16SrDNA gene were determined by qPCR and bacteria of the respective sample were isolated on ampicillin selective agar plates, followed by the determination of the species and phenotypic resistance profiles. The relative prevalence of class 1 integrons in relation to 16SrDNA was calculated and correlated to phenotypic resistance. Our findings show a high relative prevalence of class 1 integrons in water reticulation systems of household environments and in particular shower U-bends. Furthermore, prevalence of class 1 integrons correlates strongly (r_s_ = 0.95) with total phenotypic resistance at a sample point and suggest that a standardized assay determining the relative prevalence of class 1 integrons could be used as a useful screening tool in the assessment of multi-resistance in environmental samples.

## Introduction

One of the biggest challenges of health care systems worldwide is the increasing number of pathogenic bacteria resistant to a growing number of antibiotics. This process is even more critical since the number of new antibiotics developed, decreased from 19 between 1980 and 1984 to six between 2010 and 2014 [1]. *Pseudomonas aeruginosa* for example is a common cause for bloodstream, urinary tract, and surgical-site infections. In the United States 13% of health care–associated *P*. *aeruginosa* infections per year are multidrug resistant and 400 deaths per year are attributed to these infections [2]. Enterobacteriaceae resistant to cephalosporin antibiotics are responsible for 1700 deaths per year of health care–associated infections and some extended spectrum ß-lactamase (ESBL)-producing Enterobacteriaceae are resistant to nearly all antibiotics of the penicillin and cephalosporin classes [2]. This list of bacteria resistant to even the latest generation of antibiotics is growing. Causes of this antibiotic resistant crisis are the overuse and inappropriate prescription of antibiotics in healthcare and the extensive use as growth supplements in livestock [1]. Furthermore a role of biocides in this process has been discussed. Mechanisms by which biocides can effect antibiotic resistance are commonalities of target sites between biocides and antibiotics that lead to a selection of mutants and the emergence of cross-resistance, the co-selection and persistence of antibiotic-resistant strains by sub-inhibitory concentrations of biocides and antiseptics as well as the primary antibiotic and the selection of multidrug-resistant strains through polygamous mechanisms such as efflux pumps [3].

The efflux pumps *qacE* and its attenuated form *qacEΔ1* for example are located on class 1 integrons and are responsible for resistance of bacteria to quaternary ammonium compounds (QAC) including biocides like benzalkonium chloride [4,5].

Integrons are part of mobile genetic elements and have the ability to capture, integrate and express gene cassettes with minimal disturbance to the existing microbial genome. The gene cassettes are integrated and expressed via a common promoter and are often embedded in plasmids and transposons, facilitating their lateral transfer into a wide range of bacteria. They can confer several phenotypes including resistance to a broad range of antibiotic classes, heavy metals and biocides [5,6,7]. This makes a co-selection of biocide and antibiotic resistance likely. Class 1 integrons have been commonly reported among clinical bacteria [8], are linked to common resistance genes, have penetrated pathogenic and commensal bacteria, rapidly change prevalence under environmental pressure and its clinical form is xenogenetic [9,10]. The prevalence of the class 1 integron-integrase gene (*intI1*) per *16S rDNA* copy has been identified as a proxy for anthropogenic impact and has already been used as a marker for AMR gene dissemination. Furthermore a key role of quaternary ammonium compounds (QACs) in the selection for class 1 integrons was proposed. In addition to biocides, detergents can add to the selective pressure and thus to the selection of class 1 integrons [4,5,9,11].

Cleaning/disinfecting and personal care products are omnipresent in households as well as public and clinical environments and contain a broad spectrum of detergents and biocides. Furthermore, the current trend for washing at low temperatures has led to the incorporation of antimicrobial compounds into the washing process, with the use of antimicrobial products for dishwashers and washing machines also increasing [12,13]. Bacteria resistant to antimicrobials are not only present in the clinical sector but have also been detected in foods, animals and environmental areas such as soil or wastewater treatment plants [14–16]. Although the shed of AMR into the household environment is likely and the use of antibiotics, detergents and biocides can promote their selection and persistence, data on antimicrobial resistance in this area remains limited [17]. We thus hypothesized whether a high prevalence of *intI1* can be found in household environments regularly exposed to detergents and biocides and aimed to investigate the correlation of high *intI1* prevalence with the prevalence of phenotypically resistant bacteria to antibiotics based on EUCAST clinical breakpoints [18]. In case of a good correlation this prevalence could be a useful screening tool in the assessment of multi-resistance in environmental samples.

For the assessment of phenotypic resistance to biocides we determined the minimal inhibitory concentration (MIC) of benzalkonium chloride.

Benzalkonium chloride is frequently used as a preservative and disinfectant in pharmaceutical, cosmetic and hygienic formulations and Gaze *et al*. found a high incidence of class 1 integrons in QAC polluted environments [5].

Since we were interested in the correlation between the prevalence of *intI1* and bacterial resistance to clinically important antibiotics, we chose a primer combination proposed by Gillings *et al*. (2015), targeting the clinical sequence of *intI1* [9].

## Material and methods

### Sampling

All samples were collected between August and October 2018. For all samples, the owners of the land, plant or households gave permission to conduct the study on these sites. The field studies did not involve endangered or protected species.

Kitchen sink U-bends, bathroom sink U-bends, dishwasher, washing machines and toothbrushes of 28 households from the district of Kleve (Germany) were probed. Samples were collected by probing the surfaces of the inner tubing of U-bends, the sumps and sieves of dishwashers or the detergent trays of washing machines by swab sampling. The number of toothbrush samples per household varied due to the different number of residents.

Sewage sludge from a wastewater treatment plant (WWTP) and non-contaminated soil samples from organic farm fields (used for plant breeding; fertilized with organic manure only) in Kleve, served as high and low prevalence controls. Sewage sludge is a reservoir of various biocides, antibiotics and other chemicals and has been shown to be a hotspot of *intI1* prevalence and contains a large array of resistant bacteria [11,16,19,20]. Non contaminated soil samples exhibit a rather low prevalence of *intI1* and multi-resistant bacteria are less frequent [11, 21]. Sewage sludge samples were collected by transferring the material directly into a sterile collection tube using a sterile spoon or pipette. Soil samples were collected at plough depth level (0–15 cm) using a spate and sterile spoon.

### DNA extraction

Biofilms collected by swab sampling were suspended in 500 μL of sterile 0.9% sodium chloride (three swabs were combined). The toothbrush heads were cut off and biofilms were released by vigorous vortexing in 15 mL of sterile 0.9% sodium chloride for 1 min. After centrifugation at 4000g/8°C/30 min the pellet was re-suspended in 500 μL of sterile 0.9% sodium chloride. Soil samples were weighed to 1g and suspended in 10 mL of sterile 0.9% sodium chloride. Sewage sludge samples were collected from the WWTP in Kleve, Germany. 1 g of sludge sample was centrifuged at 4000g/8°C/10 min and the resulting pellet was re-suspended in 10 mL of sterile 0.9% sodium chloride.

For purification of total DNA, Fast DNA Spin Kit for Soil (MP Bio, Santa Ana, CA, USA) was used according to the manufacturer’s instructions with the exception of applying 250 μL of suspended sample instead of 500 mg solid material.

### qPCR

For determination of 16S ribosomal DNA (*16S rDNA*) and *intI1* genes, qPCR was performed. 1 μL of purified sample DNA, standards, low prevalence and high prevalence controls or non-template control (qPCR grade water) was applied to 19 μL of master mix consisting of 10 μL FastStart Essential DNA Green Master (Roche Life Sciences, Mannheim, Germany), 8.6 μL of PCR grade water and 0.2 μL of 10μM forward and reverse primer. qPCR was performed on LightCycler 480 (Roche Life Sciences, Mannheim, Germany) using the following parameters. 95°C/10 min initial activation and denaturation, 35 cycles (95°C/15 sec denaturation; 68°C/15 sec annealing; 72°C/15 sec extension), 72°C/90 sec final extension, and final melting curve analysis from 60°C to 95°C at a rate of 5 sec/1°C. Amplicons were run on agarose gels for qualitative assessment. In case of negative results 1/10 and 1/100 dilutions of the sample were analyzed to avoid false negative results due to inhibitors.

Amplicons of *intI1* and *16S rDNA* from *Pseudomonas aeruginosa* served as standards. Copies/mL of *intI1* and *16S rDNA* were determined in the same test run but separate wells. For the determination of *intI1* the primers F165 (5`CGAACGAGTGGCGGAGGGTG`3) and R476 (5`TACCCGAGAGCTTGGCACCCA`3) were used [9]. For the determination of *16S rDNA* gene the primers F919 (5´GAATTGACGGGGGCCCGCACAAG´3) and R1378 (5´CGGTGTGTACAAGGCCCGGGAACG´3) were used [22].

### Calculation of relative prevalence of *intI1*

Based on the qPCR results the relative prevalence in % (RP) of *intI1* was determined according to the following equitation.

$$RP=\left(\frac{copies\;per\;mL\;of\;intI1}{copies\;per\;ml\;of\;16S\;rDNA}\right)*2.5*100$$

For our calculations we chose an average of 2.5 copies of the *16S rDNA* per bacterial cell [11]. This value is a matter for discussion, because other studies use a value of four copies [21]. This should be considered when comparing studies that used different copy numbers for the *16S rDNA*.

### Isolation of bacteria

Bacteria of selected samples (n = 17) with *intI1* prevalence across the full spectrum of RPs (0–25.14), were isolated on TSA plates (Merck, Darmstadt, Germany) supplemented with 100 μg/mL of ampicillin (Sigma Aldrich, Munich, Germany). The pre-selection of isolates by ampicillin was meant to lower the complexity and to increase the likelihood for isolation of multi-resistant phenotypes within the usually overwhelming number of non-resistant bacteria in a sample. We adopted this method form *Ash et al*. (2002) who used ampicillin to select for potential β-lactamase producers and found that ampicillin resistance and other resistance genes, were identified in 70% of the plasmids isolated [23].

Samples were spread plated by application of 100 μL of sample and incubated for 48 h at 30°C. After incubation colonies were separated into groups based on colony morphology. Of each group one isolate was picked and sub-cultured. After sub-cultivation for 24 h at 30°C, pure isolates were applied to *VITEK 2 System* for the determination of species and resistance.

In total 91 isolates were analyzed.

### Determination of species, antibiotic resistance and corresponding MIC values

After 24 h incubation on TSA at 30°C, fresh isolates were applied to *VITEK 2 compact* System (bioMérieux, Nürtingen, Germany) for the determination of bacterial species and resistance to antibiotics (ampicillin, piperacillin/tazobactam, cefuroxime, cefuroxime/axetil, cefpodoxime, cefotaxime, ceftazidime, imipenem, meropenem, gentamicin, ciprofloxacin/fluorquinolone, tetracycline, trimethoprim/sulfamethoxazole), according to the manufacturer’s instructions. Clinical resistance was assessed based on *EUCAST* breakpoints. *VITEK 2 GN* and *VITEK 2 AST N-215* were used for the identification and determination of antibiotic resistance of gram-negative bacteria, without prior determination of gram status.

### Determination of MIC of benzalkonium chloride

Isolates were sub-cultured in tryptic soy broth (Merck, Darmstadt, Germany) for 24 h at 30°C/150 rpm. The minimal inhibitory concentration (MIC) of benzalkonium chloride (Sigma Aldrich, Munich, Germany) was determined by resazurin assay as previously described [24].

### Statistics

For statistical analysis, samples were grouped from lowest to highest prevalence as follows: Group 1 (RP <0.001; median 0.0), group 2 (RP <0.3; median 0.01), group 3 (RP 0.3–4.2; median 1.99) and group 4 (RP 4.5–25.1; median 8.15). This was done to simulate different cut-off values for determination of a high resistance potential and increase of multi-resistant phenotypes respectively.

Statistics were performed using GraphPad Prism (GraphPad Software Inc.). Relative prevalence values of *intI1* were not normally distributed and parametric (determined by Shapiro Wilk normality test), thus prevalence values of sample areas and households were compared by Kruskal-Wallis test, followed by Dunn`s multiple comparison test. Spearman correlation was used for comparison of median *intI1* prevalence values, MIC values and phenotypic resistance.

## Results

### Distribution of *intI1* across household environments

**Fig 1:**
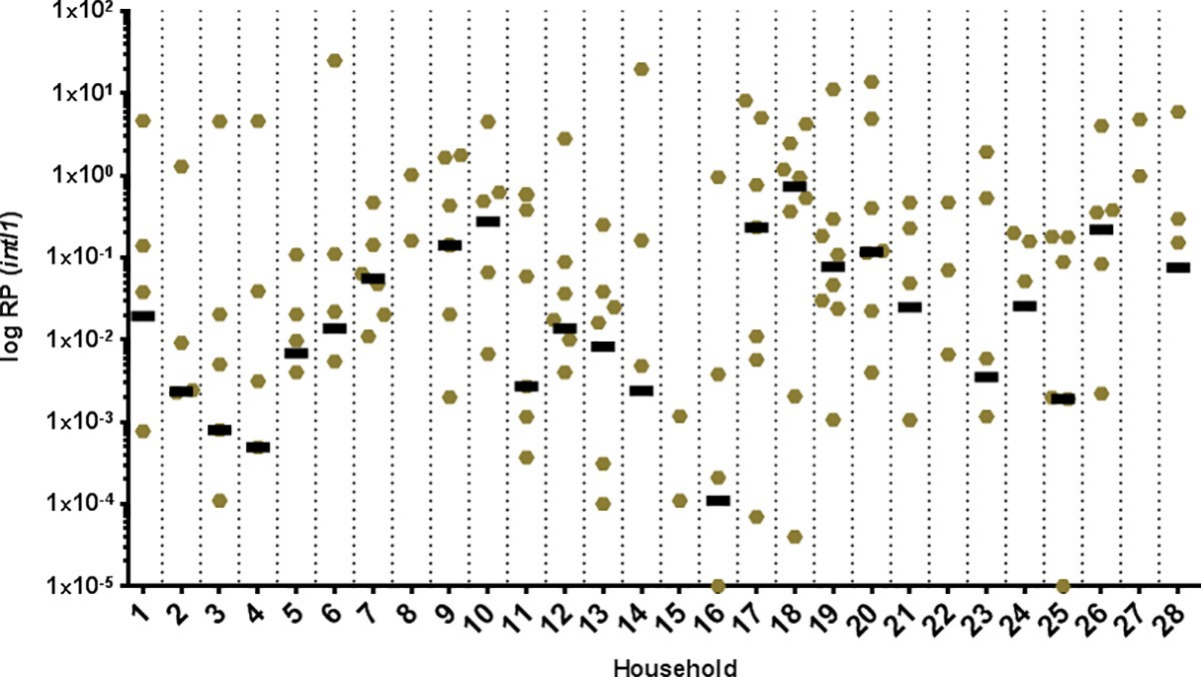
Relative prevalence (RP) of class 1 integrons across household environments. Bars depict the median. Copies/mL of *intI1* and *16S rDNA* gene were determined by qPCR and the relative prevalence of *intI1* was calculated. The median value of toothbrushes is 0 and thus not depicted.

*IntI1* is present in all sample areas (see Fig 1), indicated by exceeding prevalence values compared to the low prevalence control (soil: 0.0007). Median RP of *intI1* is highest in shower U-bends (0.27) followed by bathroom sink U-bends (0.14), household washing machines (0.008), kitchen sink U-bends (0.005), dishwasher sieves (0.002), toothbrushes (0.001) and dishwasher sumps (0.001). The median of our high prevalence control (sewage sludge) is considerably higher (11.9). Of note, median *intl1* prevalence in sewage sludge and soil corresponds well with previously described values of the literature [21].

**Fig 2:**
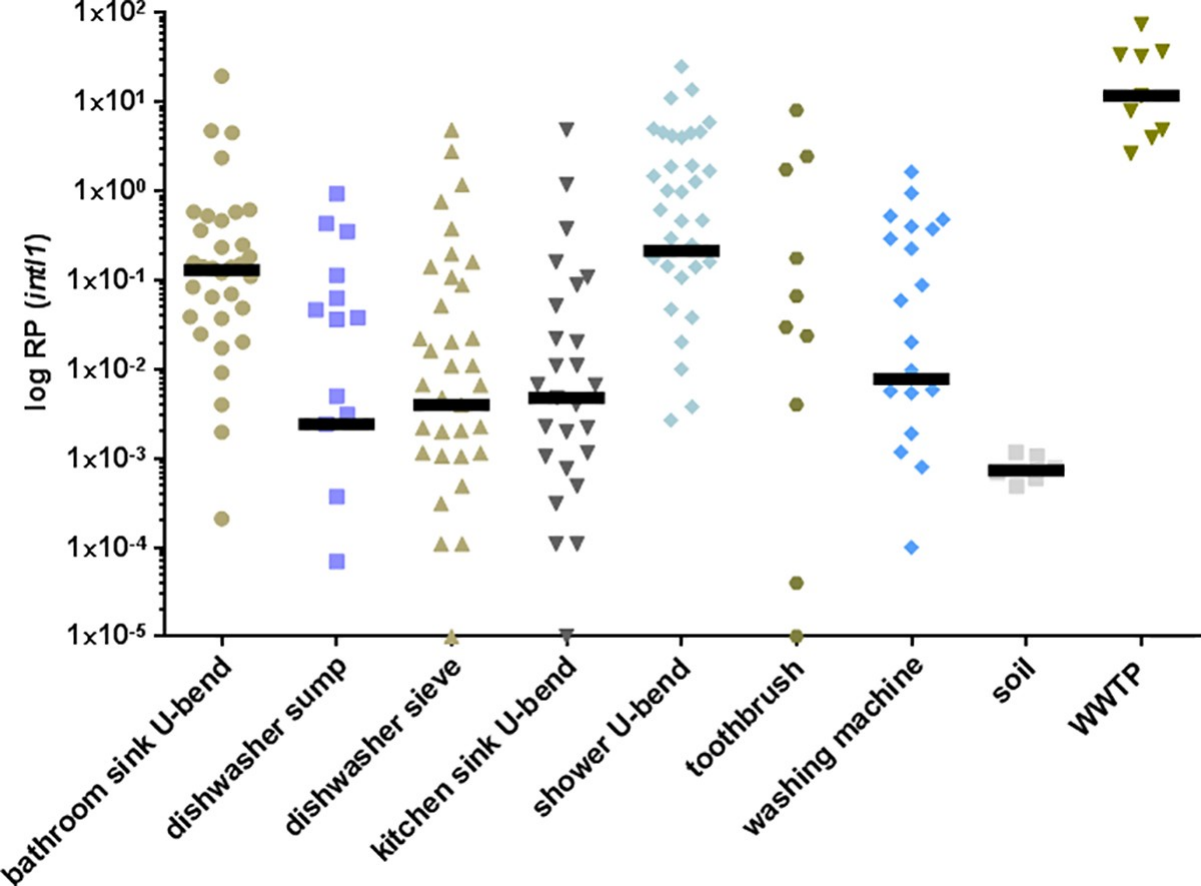
Relative prevalence (RP) of class 1 integrons across households. Bars depict the median. Copies/mL of *intI1* and *16S rDNA* gene were determined by qPCR and the relative prevalences of *intI1* was calculated. Median of households 8, 15, 22 and 27 is 0.

Kruskal-Wallis test reveals that media RP values between the different household areas vary significantly (χ2 = 66.69; p < 0.0001; df = 6). However, RP values within the same household area show a high coefficient of variation (median CV% = 152.9) and Dunn`s multiple comparison test identifies a significant difference only between bathroom sink U-bends and toothbrushes (p < 0.0001) as well as shower U-bends and toothbrushes (p < 0.0001). The distribution of *intI1* prevalence between households is diverse and Kruskal-Wallis test reveals the variation between median RP values is not significant (χ2 = 37.70; p = 0.0826; df = 27). Again the median CV% value of RPs form one household is high (177.2). See also Figs 1 and 2.

### Correlation between *intI1* prevalence and bacterial resistance

**Fig 3:**
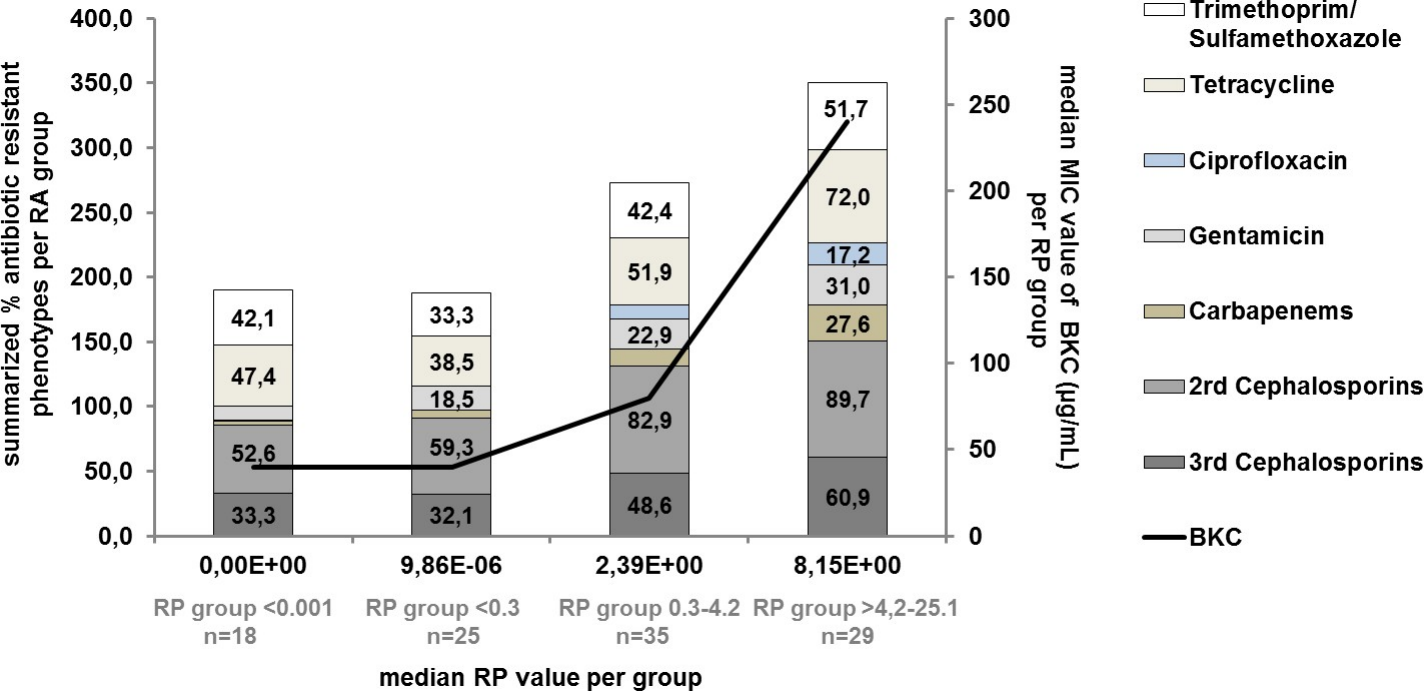
Correlation between median prevalence (RP) of *intI1* and resistant phenotypes per RP group. The left Y-axis depicts the summarized % of antibiotic resistant phenotypes per RP group, the right Y-axis depicts the median MIC value of BKC (*μ*g/mL) per RP group. The black line depicts the median of MIC values to benzalkonium chloride (BKC). Spearman correlation between median RP of *intI1* and resistant phenotypes per RP group was found at 0.95. Numbers in stacked bars are % of resistant phenotypes to the respective antibiotic. Please see method section for RP groups.

**Fig 4:**
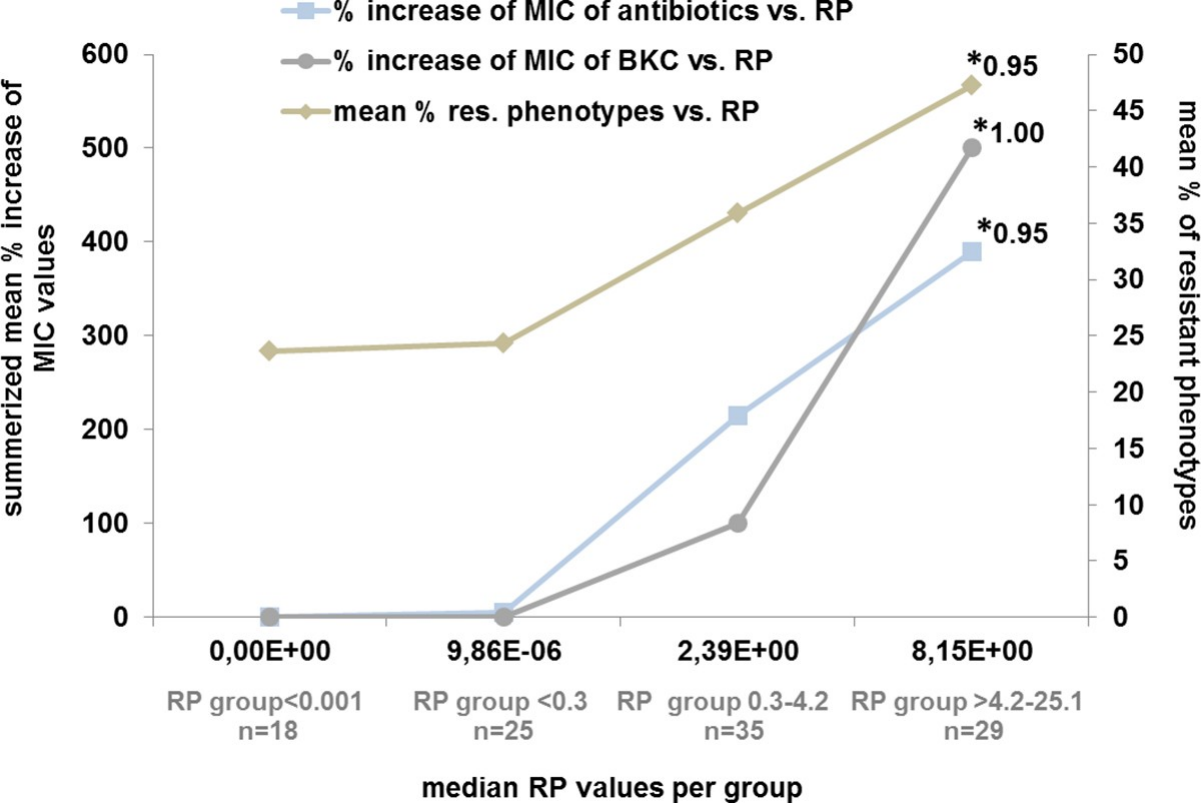
Correlation of *intI1* prevalence (RP) vs. increase of MIC to antibiotics and benzalkonium chloride (BKC) and percentage of resistant phenotypes per RP group. The left Y-axis depicts the summarized mean % increase of MIC value per RP group, the right Y-axis depicts mean % of resistant phenotypes per group. * Spearman correlation. Please see method section for RP groups.

Regarding the prevalence of *intI1* and the number of corresponding antibiotic resistant phenotypes, a strong Spearman correlation of 0.95 is found across all bacterial isolates (see Figs 3 and 4).

Between group 1 (RP <0.001; median 0.0) and group 4 (RP 4.5–25.1; median 8.15) the number of phenotypes resistant to 2^nd^ and 3^rd^ generation cephalosporins goes up (+37% and +27.6%). Resistance to gentamicin (+20.5%) and carbapenems (imipenem +22% and meropenem +27.6%) show a comparable increase followed by resistance to ciprofloxacin/fluoroquinolone (+17.2%), tetracycline (+24.6%) and trimethoprim/sulfamethoxazole (+9.6%). Ciprofloxacin/fluoroquinolone resistant phenotypes are only found in areas with a median RP of >0.5 while ceftazidime resistant phenotypes are absent in areas with a median RP of <0.001. The overall rise in MIC values of antibiotics correlates well with RP values, as shown in Fig 4 (Spearman correlation of 0.95).

Furthermore, the MIC of benzalkonium chloride correlates strongly with the number of resistant phenotypes (Spearman correlation of 1.00). The median MIC of benzalkonium chloride of RP groups <0.3, 0.3–4.2 and 4.5–13.5 increases from 40 μg/mL, 80 μg/mL to 240 μg/mL. MIC values of >640 μg/mL are only found in *P*. *aeruginosa*, *Pseudomonas putida*, *Pseudomonas fluorescens* and *St*. *maltophilia* at RP values >0.96. These species are prone to an increased tolerance towards biocides and *Mc Cay et al*. (2010) for example show that *P*. *aeruginosa* can be adapted to higher MICs in the presence of subinhibitory concentrations of benzalkonium chloride. Here MIC values increase from 25 μg/mL for the wild-type strain (NCIMB 10421), to >350 μg/mL after serial passage and efflux-pump activity is the main contributor in this process [25].

### Distribution of bacterial species

**Fig 5:**
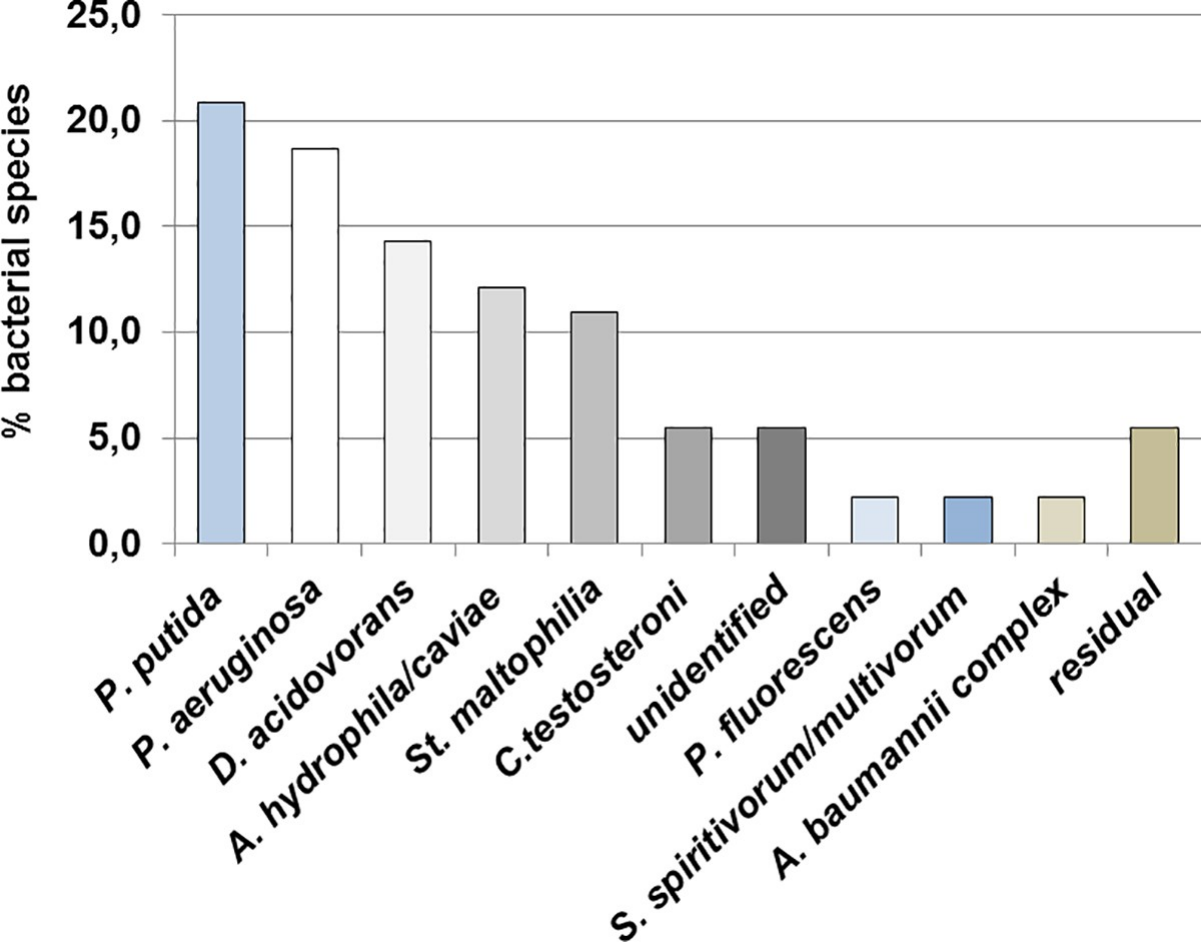
Bacterial species in %, isolated from household environments on ampicillin-selective TSA plates. Species were determined by *VITEK 2* system (bioMérieux).

The relative distribution of bacterial species is shown in Fig 5. The most abundant species across all isolates (n = 91) are *P*. *putida* (20.9%) and *P*. *aeruginosa* (18.7%), followed by *Delftia acidovorans* (14.3%), *Aeromonas hydrophila/caviae* (12.1%), *St*. *maltophilia* (11%), *Comamonas testosteroni* (5.5%), *P*. *fluorescens* (2.2%), *Acinetobacter baumannii complex* (2.2%) and *Sphingobacterium spiritivorum/multivorum* (2.2%). 5.5% of the isolates could not be identified and the residual group (5.5%) containes *Chryseobacterium indologenes*, *Morganella morganii ssp morganii*, *Serratia marcescens*, *Pseudomonas mendocina* and *Enterobacter cloacae complex* (each 1.1%). Of note, bacterial species are distributed equally among the different sample areas and no preference of certain bacterial species for specific household environments is observed. However, bacteria prone to multi-resistance mainly occur in environments with a high prevalence of *intI1*.

### Correlation of *intI1* prevalence and the occurrence of multi-resistant bacteria

**Fig 6:**
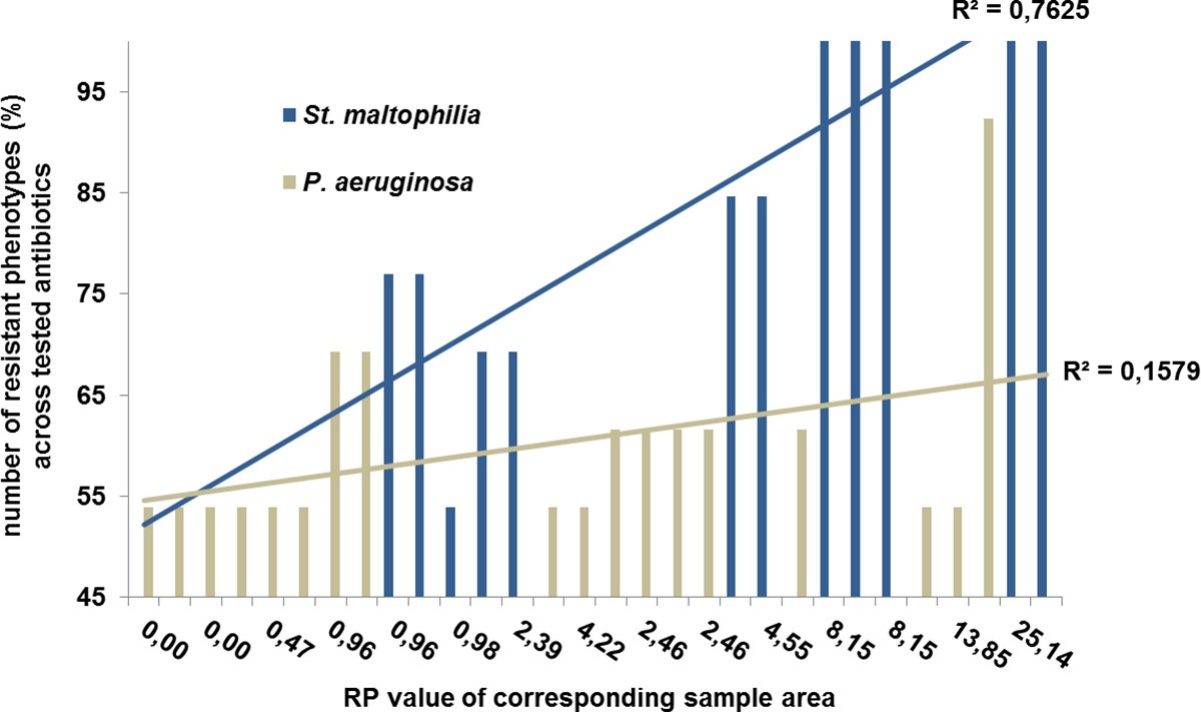
Phenotypic resistance of *St. maltophilia* and *P. aeruginosa* isolates in relation to increasing *intI1* prevalence (RP) of the sample area.

In Fig 3, a strong correlation between the prevalence of *intI1* and the number of resistant phenotypes of a sample is found. However, bacteria resistant to all tested antibiotics are only present in sample areas with RP values ≥8.15 (range: 8.15–25.14). In this group (n = 19) 26% of isolates are resistant to all tested antibiotics. These truly multi-resistant bacteria, *St*. *maltophilia* (n = 3), *C*. *indologenes (n = 1)* and *P*. *aeruginosa* (n = 1) are all found in locations with the most intimate connections to humans (showers, toothbrushes and bathroom sinks). The phenotypic resistance of *St*. *maltophilia* isolates increases with rising RP values of the respective sample area while *P*. *aeruginosa*, being equally prone to multiple resistances, is less affected by an increased *intI1* prevalence (see Fig 6).

## Discussion

Previous studies established a link between the use of detergents and biocides (in particular QACs) and the occurrence of class 1 integrons. Furthermore class 1 integrons and resistant bacteria have been described to be present in the influx and efflux of WWTP and river sediments [4,5,19] but no studies on their distribution in households, particularly in relation to the simultaneous occurrence of phenotypic resistant and multi-resistant bacteria, are present. It has also been shown that washing machines, dishwashers and U-bends provide an aqueous environment with constant nutrient supply which promotes bacterial growth and the formation of biofilms [26,27]. Moreover, these environments have a substantial influx of bacteria from humans, animals or food origin, as well as biocides and detergents from cleaning, disinfecting and personal care products. Biocides and Detergents might create a highly selective environment for resistant bacteria and could promote their persistence.

The current study had two major objectives. The first was to investigate the prevalence of class 1 integrons in household environments using a primer pair targeting the clinical version of *intI1* previously proposed by Gillings *et al*. (2015), followed by the calculation of the ratio of *intI1* per *16S rDNA* copy (relative prevalence of *intI1 = RP*). The second objective was to identify the number of resistant phenotypes to antibiotics as well as the MIC values to benzalkonium chloride of isolates. This was followed by the correlation of phenotypic resistance data with the respective RP values of the sample point. Although we used ampicillin selective tryptic soy agar for isolation of resistant phenotypes, this should not bias the results. We aimed to compare the relative prevalence of class 1 integrons to the prevalence of resistant and multi-resistant bacteria and this technique increased the likelihood of isolation even in samples with a low fraction of resistance. If anything, this should lower the proposed positive correlation of RP and resistance rather than promote it.

The results show a widespread presence of class 1 integrons in all household samples and its prevalence at a certain sample area strongly correlates with the occurrence of multi-resistant bacteria. We thus suggest the use of the relative prevalence of class 1 integrons as a marker for a high phenotypic resistance.

### Class 1 integrons in household environments

The obtained results show a high variation of *intI1* prevalence between samples of the same household area but of different households, ranging from e.g. 0 to 25.14 in case of shower U-bends. Thus the median prevalences of *intI1* of household areas are considerably lower than the median prevalence of *intI1* of sewage sludge (11.9), which we used as our high prevalence control. However, in most cases median *intI1* prevalence of household areas was higher than our low prevalence control (soil).

In particular, shower and bathroom sink U-bends are hotspots of class 1 integrons and also other studies prove the presence of *intI1* genes in biofilms and environmental water [4,19,28]. This may indicate the shed of these strains form humans and/or the selective accumulation through the strong use of antibacterials/biocides/detergents in shower gels, toothpastes, etc. This is also supported by a study of Marshall *et al*. (2012) that showed a trend towards higher resistance levels in some household areas where biocides are used [29]. Furthermore, the occurrence of *intI1* genes correlates with QAC occurrence [5] and correlates with high MIC values to benzalkonium chloride in this study as well.

### Distribution of bacterial species across households

The most prominent species found in this study are *P*. *putida* and *P*. *aeruginosa*. Both bacteria are residents of aquatic systems and have been detected in other studies in the domestic environment [26,27]. Wild-type strains are known to carry multiple resistances [30,31] and class 1 integrons are associated with both species [32]. *St*. *maltophilia*, *D*. *acidovorans* and *A*. *hydrophila/caviae* are also found in high numbers. *P*. *fluorescens*, *Comamonas testosteroni*, *S*. *multivorum*, *S*. *spiritivorum* and *A*. *baumannii* complex are found less frequently. In general, most of the identified species are regarded opportunistic pathogens and have been associated with AMR. In particular *Pseudomonas*, *Stenotrophomonas* and *Acinetobacter are* associated with waterborne infections due to their presence in biofilms of water reticulation systems [33–38].

Bacterial species found in this study are distributed equally among the different sample areas and no preference of certain bacterial species for specific household environments is observed. However, species prone to multi-resistance (e.g. *St*. *maltophilia)* are found in environments with high RP values. The low variation of species in different household areas and households may be due to the same environmental conditions concerning e.g. nutrients, temperature and pH or to the selective pressure of antimicrobials in e.g. cleaning and hygienic products. In this respect home care might be of great concern, since multi-resistant bacteria can cause serious infectious and might lead to complications in elderly people.

### Correlation of *intI1* prevalence and multi-resistant bacteria

A closer look at the RP values of a certain sample point and the prevalence of resistant bacteria reveals a strong correlation between *intI1* prevalence and the number of resistant phenotypes of the respective sample. The most prominent bacterial species in samples with high RP values is *St*. *maltophilia* and phenotypic resistance of this strain increases with increasing *intI1* prevalence. However, *P*. *aeruginosa* which is equally prone to AMR did not change its resistance profile at higher RP values like *St*. *maltophilia*. Studies revealed that trimethoprim/sulfamethoxazole resistance of *St*. *maltophilia* is associated with class 1 integrons [37,38] and the increase of resistance towards these antibiotics at rising RP values corresponds to this finding. Pseudomonads tend to over-produce chromosomal ampC-β-lactamases [32], which might be related to the high level of AMR even at low RP values.

Amos *et al*. found quaternary ammonium compounds resistance genes (qacE and qacH) in 75% of class 1 integrons [4]. The correlation of *intI1* prevalence and high MIC values to benzalkonium chloride in our study corresponds well with this.

At high prevalence values the number of resistant phenotypes to all tested antibiotics increases. Most resistance genes in Enterobacteriaceae are located on class 1 integrons and have been analyzed *inter alia* in *Pseudomonas*, *Aeromonas and Stenotrophomonas* {Formatting Citation} making a correlation of *intI1* prevalence and resistant species reasonable. Particularly resistance towards critically important antibiotics, like 2^nd^ and 3^rd^ generation cephalosporins as well as carbapenems (imipenem and meropenem) and gentamicin, increases substantially.

Increasing resistances towards cephalosporins might partly be caused by the expression of ampC-β-lactamases. Enzymes such as ampC-β-lactamases are often plasmid-borne and hydrolyze penicillins, 2^nd^ and 3^rd^ generation cephalosporins and monobactams [39]. However, to correlate the relative prevalence of class 1 integrons with cephalosporinases, further analysis of resistance genes and *intI1* is required regarding all groups of β-lactamases.

### Prevalence of *intI1* as a screening tool for assessment of multi-resistance and determination of anthropogenic pollution

A large number of studies shows that the prevalence of *intI1* can be used for the assessment of AMR gene load and the level of anthropogenic pollution [4,5,6,9,10,11]. Regarding these results and the results of our study, it is possible to design a screening test for the prediction of the resistance potential and probably the occurrence of multi-resistant strains of a sample, based on the prevalence of *intI1*. However, most studies determining *intI1* use different primer pairs, standards and calculation methods and thus comparison of studies is difficult. This means, for such an assay to be relevant, more studies using a standardized test are mandatory assessing different environmental areas. For an assessment and validation, particularly water reticulation systems in non-clinical and clinical environments would be of interest, because Herruzo *et al*. (2017) shows that bathroom sink U-bends can be a reservoir of OXA-48 *K*. *pneumoniae* and are linked to a colonized patient’s stay [40]. Furthermore the persistence of the bacteria, as a reservoir, may contribute to the perpetuation of an outbreak in hospital patients.
